# The resistance risk of fluopicolide and resistance-associated point mutations in the target protein PlVHA-a in *Phytophthora litchii*

**DOI:** 10.1007/s44154-025-00218-9

**Published:** 2025-04-21

**Authors:** Tan Dai, Jikun Yang, Shiping Hu, Chuang Zhao, Kang Yuan, Jianqiang Miao, Xili Liu

**Affiliations:** 1 State Key Laboratory for Crop Stress Resistance and High-Efficiency Production, College of Plant Protection, Northwest A&F University, Yangling , Shaanxi, 712100 China; 2 Department of Plant Pathology, College of Plant Protection, China Agricultural University, 2 Yuanmingyuanxi Road, Beijing, 100193 China

**Keywords:** Resistance mechanism, PlVHA-a, *Phytophthora litchii*, Fluopicolide

## Abstract

Litchi, a fruit that is highly sought-after worldwide, faces significant yield challenges due to litchi downy blight, primarily caused by *Phytophthora litchii*. Fluopicolide has exhibited remarkable efficacy in inhibiting this pathogen and is utilized for the management of litchi downy blight. Although understanding the resistance of *P. litchii* to fluopicolide is critical, studies on its risk and mechanisms remain limited. In this study, we determined the sensitivity of 125 *P. litchii* isolates to fluopicolide, revealing an average EC_50_ value of 0.131 ± 0.037 μg/mL. Through fungicide adaptation, four resistant mutants were obtained with resistance factors exceeding 600, indicating that these strains exhibited high levels of resistance. A compound fitness index analysis demonstrated that the survival fitness of resistant mutants was significantly lower than that of their parental strains. Cross-resistance assays revealed no cross-resistance between fluopicolide and other fungicides with different modes of action. However, positive cross-resistance was observed with fluopimomide. A comprehensive evaluation suggested a moderate risk of *P. litchii* developing resistance to fluopicolide. PlVHA-a^N771S^ and PlVHA-a^N846S^ point mutations in resistant mutants were identified by gene sequencing analyses. These two point mutations were validated as contributors to resistance in *P*. *litchii* through genetic transformation and molecular docking.

**Supplementary Information:**

The online version contains supplementary material available at 10.1007/s44154-025-00218-9.

## Highlights

The baseline sensitivity of *Phytophthora litchii* to fluopicolide was established.

*P. ltichii* presents a moderate resistance risk to fluopicolide.

The point mutation N771S or N846S in PlVHA-a could confer fluopicolide-resistance in *P. litchii*.

**Supplementary Information:**

The online version contains supplementary material available at 10.1007/s44154-025-00218-9.

## Introduction

Litchi, an evergreen subtropical fruit tree belonging to the family Sapindaceae, is indigenous to China and primarily cultivated in Guangdong, Guangxi, Fujian, and Hainan provinces. Its skin is red and visually appealing, while its flesh is white and translucent with a sweet, juicy flavor that is both soft and crisp. It is popular among consumers worldwide (Jiang et al. 2006).

The plant pathogens infecting litchi include *Phytophthora litchii* (Zheng et al. 2019), *Colletotrichum siamense* (Ling et al. 2019), *Geotrichum candidum* (Yu et al. 2023), and *Fusarium incarnatum* (Gao et al. 2021). Notably, the oomycete *P. litchii* causes litchi downy blight (LPB), the most common disease affecting litchi yield. *P. litchii* predominantly targets near-mature fruit but can also affect young fruit, fruit stalks, flower spikes, and branches bearing fruit. Under prolonged rainy conditions coupled with suitable temperatures, it can also induce leaf diseases. *P. litchii* can lead to significant fruit drop during the growth phase as well as browning or even rotting of fruit during post-harvest storage and transportation, thereby diminishing the commercial value of fruit (Liu et al. 2005). However, oomycete diseases often cannot be effectively managed using antifungal compounds due to physiological and biochemical distinctions between oomycetes and fungi, including cell wall components, chromosome ploidy levels, and membrane sterol composition (Ishii and Holloman 2015; Rossman and Palm 2006; Tang et al. 2021). Fungicides commonly used to control LPB include fluopicolide (V-ATPase inhibitor), mancozeb (multi-site inhibitor), metalaxyl (RNA polymerase I inhibitor), pyraclostrobin (quinone outside inhibitor), carboxylic acid amides (cellulose synthase inhibitors), oxathiapiprolin (oxysterol binding protein homologue inhibitor), and cyazofamid (quinone inside inhibitor) (http://www.chinapesticide.org.cn/).

Fluopicolide, a pyridinylmethyl-benzamide fungicide developed by Bayer Crop Science, exhibits exceptional protective properties along with curative efficacy against plant oomycete diseases while maintaining a prolonged field duration. The chemical designation for fluopicolide is 2,6-dichloro-N-[[3-chloro-5-(trifluoromethyl)−2-pyridinyl]methyl]. This fungicide demonstrates robust inhibitory activity against phytopathogenic oomycetes relevant to agriculture such as *Phytophthora infestans* (Wang et al. 2014), *Phytophthora nicotianae* (Liu et al. 2024), *Phytophthora capsici* (Wu et al. 2020), *Pseudoperonospora cubensis* (Thomas et al. 2018), and *Phytophthora erythroseptica* (Zhang et al. 2019). Though initially regarded as a delocalization of spectrin-like protein inhibitor (Toquin et al. 2019), recent investigations have identified V-ATPase subunit a as its target protein, thus establishing it as a fungicide characterized by a unique mode of action (Dai et al. 2024a).

Assessing fungicide resistance and its mechanism is crucial for effective application and management. However, the resistance risk and mechanism of *P. litchii* to fluopicolide remain inadequately understood. Therefore, this study aimed to (i) establish a baseline sensitivity for *P. litchii* to fluopicolide, (ii) generate fluopicolide-resistant mutants and evaluate the associated resistance risk in *P. litchii*, and (iii) elucidate the resistance mechanism of *P. litchii* to fluopicolide.

## Results

### Baseline sensitivity of *P. litchii* to fluopicolide

**Fig. 1:**
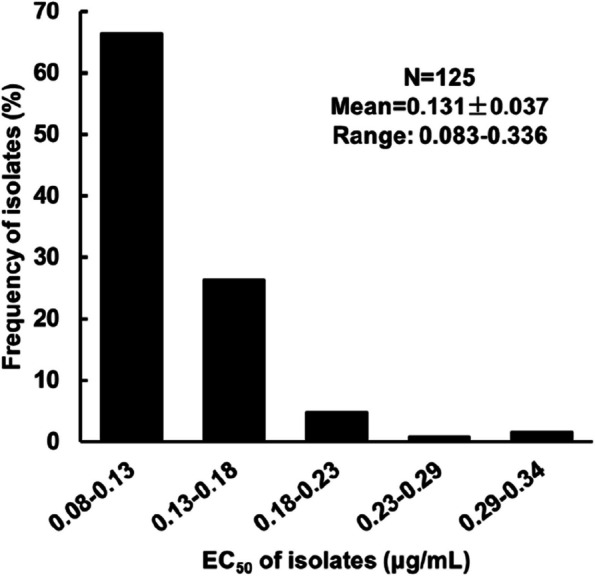
The distribution of fluopicolide sensitivity of 125 *Phytophthora litchii* isolates

The EC_50_ values of the 125 *P. litchii* isolates ranged from 0.083 to 0.336 μg/mL, with an average of 0.131 μg/mL. The distribution of EC_50_ values followed a unimodal curve (Fig. 1), indicating that there were no fluopicolide-resistant subpopulations in the field.

### Generation of fluopicolide-resistant mutant and its resistance stability

Four fluopicolide-resistant mutants, RG6-2, RG6-3, RF1-1, and RF1-2, were obtained by fungicide adaptation from two parent strains, GDHZ-6 and FJZZ-1, with a mutation frequency of 1.0 × 10^–4^. After transferring ten generations of these resistant mutants onto fungicide-free V8 medium, the resistance factors (RF) were all found to be greater than 600 (Table 1), indicating that the resistance of these mutants could be stably inherited.

**Table 1:** Characteristics of stability and resistance in fluopicolide-resistant mutants of *Phytophthora litchii.* ^y^EC_50_, median effective concentration; ^z^RF, resistance factor = EC_50_ of resistant mutant / EC_50_ of the paretal isolate

**Isolate**	**Origin**	**EC**_**50**_**(µg/mL)** ^y^		**RF**^**z**^	
		**First**	**Tenth**	**First**	**Tenth**
GDHZ-6	Parent	0.100	0.136	-	
RG6-2	Mutant	> 100	> 100	> 1000	> 735
RG6-3	Mutant	> 100	> 100	> 1000	> 735
FJZZ-1	Parent	0.107	0.144	-	
RF1-1	Mutant	> 100	> 100	> 935	> 694
RF1-2	Mutant	> 100	> 100	> 935	> 694

### Influence of temperature on mycelial growth

All mutants and parental isolates grew best at 25–30 °C, and no strain could grow at 4 °C. RG6-2 and RG6-3 grew at a temperature that was much higher than that of their parental isolates at 25–28 °C. The growth rate of RF1-1 and RF1-2 mutants was higher than that of their parental isolate at all temperatures (Table 2).

**Table 2:** Mycelial growth of *Phytophthora litchii* mutants resistant to fluopicolide at different temperatures^z^. ^z^In each group, the same letters are followed by the Mean ± standard error, which shows that there is no significant difference (*P* < 0.05)

Isolate	Mycelial growth (mm)						
	**4°C**	**13°C**	**18°C**	**22°C**	**25°C**	**28°C**	**30°C**
GDHZ-6	-	19.5 ± 0.7 a	45.0 ± 0.6 a	49.9 ± 0.3 a	60.1 ± 0.4 b	60.8 ± 0.7 b	63.1 ± 0.7 b
RG6-2	-	20.2 ± 0.4 a	44.2 ± 1.3 ab	50.0 ± 0.6 a	62.5 ± 1.4 a	62.4 ± 1.1 a	63.7 ± 0.6 b
RG6-3	-	17.7 ± 0.7 b	43.8 ± 0.8 b	49.1 ± 0.1c	62.4 ± 1.0 a	62.5 ± 0.9 a	68.4 ± 2.7 a
FJZZ-1	-	13.8 ± 1.1 c	37.9 ± 0.5 b	44.7 ± 0.6 c	61.7 ± 0.7 c	61.1 ± 0.8 c	61.30 ± 1.0 b
RF1-1	-	23.2 ± 0.5 b	53.7 ± 0.8 a	62.2 ± 1.4 a	79.1 ± 2.1 a	82.4 ± 1.2 a	75.0 ± 2.3 a
RF1-2	-	26.2 ± 0.5 a	52.8 ± 1.0 a	57.9 ± 2.1b	75.9 ± 0.9 b	73.4 ± 1.0 b	74.9 ± 0.5 a

### Compound fitness index (CFI) analysis of fluopicolide-resistant mutants in *P. litchii*

All mutants showed significantly lower sporangia production, sporangia germination, and cystospore germination than their parental strains. The mycelial growth rate of all mutants was significantly higher than that of their parental strains. The zoospore release rate was similar between the RF1-1 and RF1-2 mutants and their parental strain; however, the RG6-2 and RG6-3 mutants showed significantly lower zoospore release rates than that of their parental strain. The lesion areas caused by the mutants RG6-2 and RG6-3 were significantly larger than that caused by their parental strain. However, the lesion areas caused by the mutants RF1-1 and RF1-2 were significantly smaller than that caused by their parental strain. Overall, the CFI levels of fluopicolide-resistant mutants were significantly lower than those of their parental strains (Table 3).

**Table 3:** Fitness of *Phytophthora litchii* parental isolates and its fluopicolide-resistant mutants^z^. ^z^CFI: Compound Fitness Index, was equal to product of the multiple traits measured for each strain. Same letter followed by mean ± standard error shows that there is no significant difference (*P* < 0.05) in same group

Isolates	Mycelial growth (mm)	Sporangium production (× 10^5^/mL)	Sporangia germination rate (%)	Zoospore release rate (%)	Cystospore germination (%)	Lesion area (mm^2^)	CFI (× 10^8^)
GDHZ-6	60.1 ± 0.4 b	186 ± 3.5 a	54.2 ± 6.9 a	74.3 ± 8.7 a	84.2 ± 4.1 a	11.22 ± 1.95 b	4.26 a
RG6-2	62.5 ± 1.4 a	139 ± 2.7 b	29.8 ± 10.5 b	68.8 ± 5.3 b	61.8 ± 6.1 b	21.52 ± 6.45 a	2.37 b
RG6-3	62.4 ± 1.0 a	152 ± 2.9 b	23.2 ± 7.9 b	68.5 ± 6.9 b	57.5 ± 7.7 b	14.65 ± 4.79 ab	1.27 b
FJZZ-1	61.7 ± 0.7 c	137 ± 3.6 a	67.3 ± 5.9 a	81.1 ± 3.3 a	88.3 ± 4.8 a	13.6 ± 3.05 a	5.56 a
RF1-1	79.1 ± 2.1 a	45 ± 0.9 b	27.0 ± 5.0 c	78.5 ± 1.9 a	33.5 ± 6.9 c	4.30 ± 0.43 b	0.11 c
RF1-2	75.9 ± 0.9 b	54 ± 1.2 b	46.7 ± 5.3 b	79.5 ± 2.7 a	44.7 ± 4.6 b	5.38 ± 1.70 b	3.66 b

### Cross-resistance

**Fig. 2:**
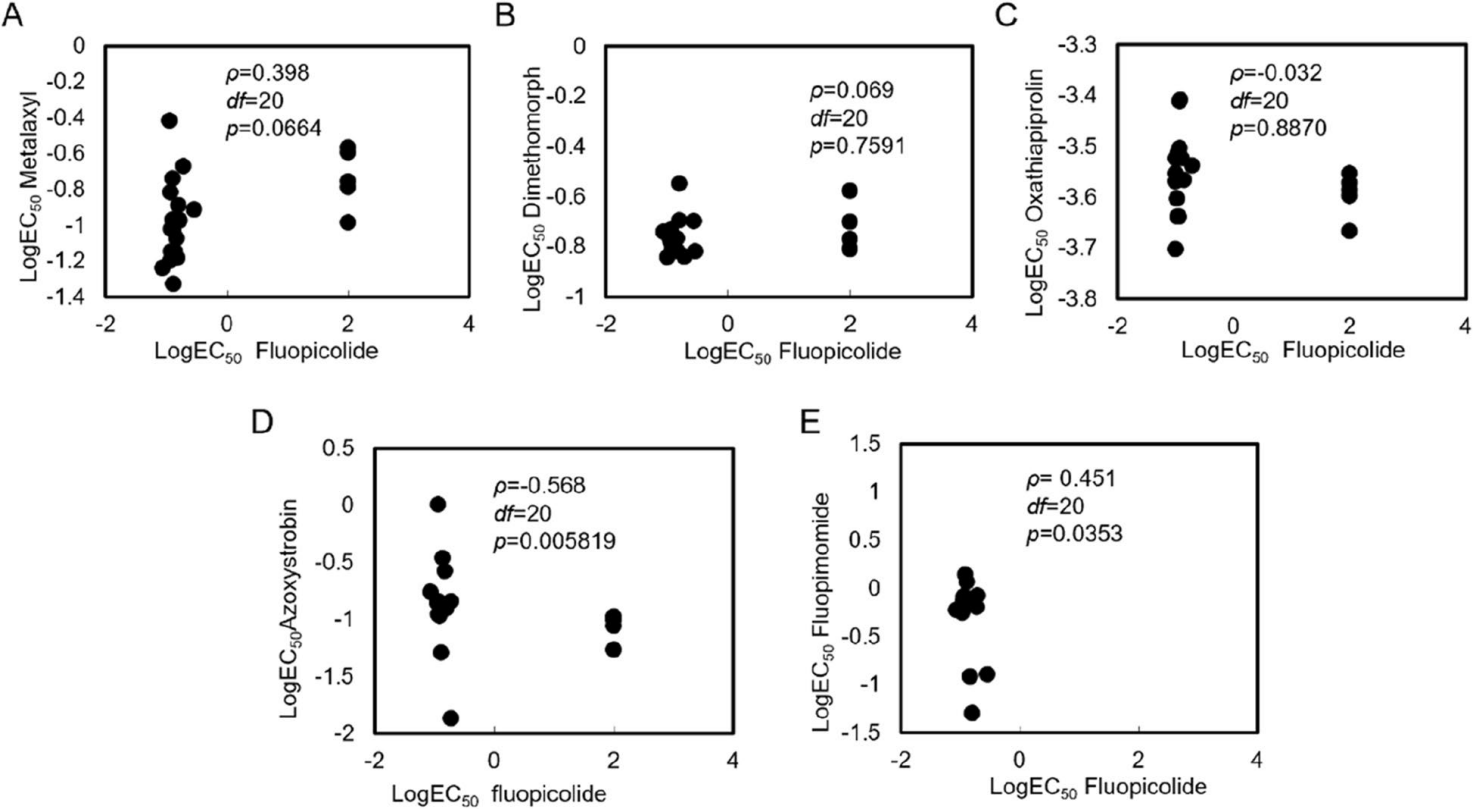
Spearman’s rank correlation coefficients for cross-resistance between fluopicolide and five other fungicides in *P. litchi.* **A** Metalaxyl, **B** Dimethomorph, **C** Oxathiapiprolin, **D** Azoxystrobin, **E** Fluopimomide

The sensitivity of resistant mutants to fluopicolide did not correlate with sensitivity to metalaxyl, dimethomorph, or oxathiapiprolin (Fig. 2A-C). However, negative cross-resistance was found between fluopicolide and azoxystrobin (Fig. 2D) and positive cross-resistance was found between fluopicolide and fluopimomide (Fig. 2E).

### Cloning and sequence analysis of *PlVHA-a*

**Fig. 3:**
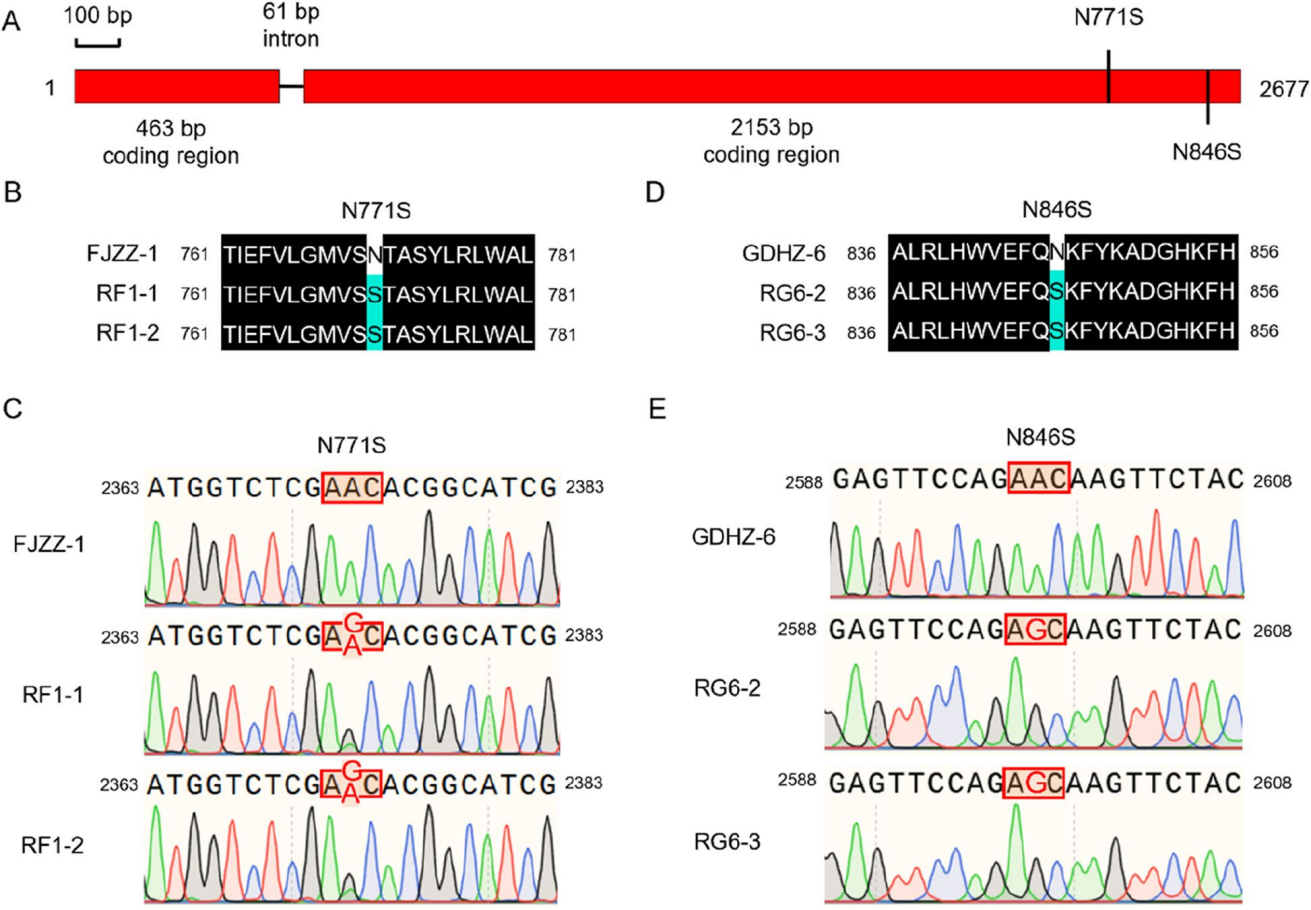
PlVHA-a protein sequence alignments of resistant mutants and their parental strains. **A** Coding regions, intron, and two point mutations N771S, and N846S of PlVHA-a gene were labeled as red boxes and black horizontal line, black vertical line, respectively. **B** The PlVHA-a protein of RF1-1 and RF1-2 contained the N771S point mutation. **C** The N771S point mutation was founded by sequencing chromatograms of *PlVHA-a*. **D** The PlVHA-a protein of RG6-2 and RG6-3 contained the N846S point mutation. **E** The N846S point mutation was founded by Sequencing chromatograms of *PlVHA-a*

The sequences of *PlVHA-a* from both the parental strains and the mutants were analyzed and compared. The findings revealed that the mutants RF1-1 and RF1-2 experienced a nucleotide transition from adenine (A) to guanine (G) at position 2373, resulting in a substitution of asparagine (N) with serine (S) at position 771 of PlVHA-a (Fig. 3B and C). Additionally, a nucleotide transition from asparagine (N) to guanine (G) at position 2598 in mutants RG6-2 and RG6-3 led to an alteration from asparagine (N) to serine (S) at position 846 of PlVHA-a (Fig. 3D and E).

### Confirmation of fluopicolide resistance-related point mutations in PlVHA-a

**Fig. 4:**
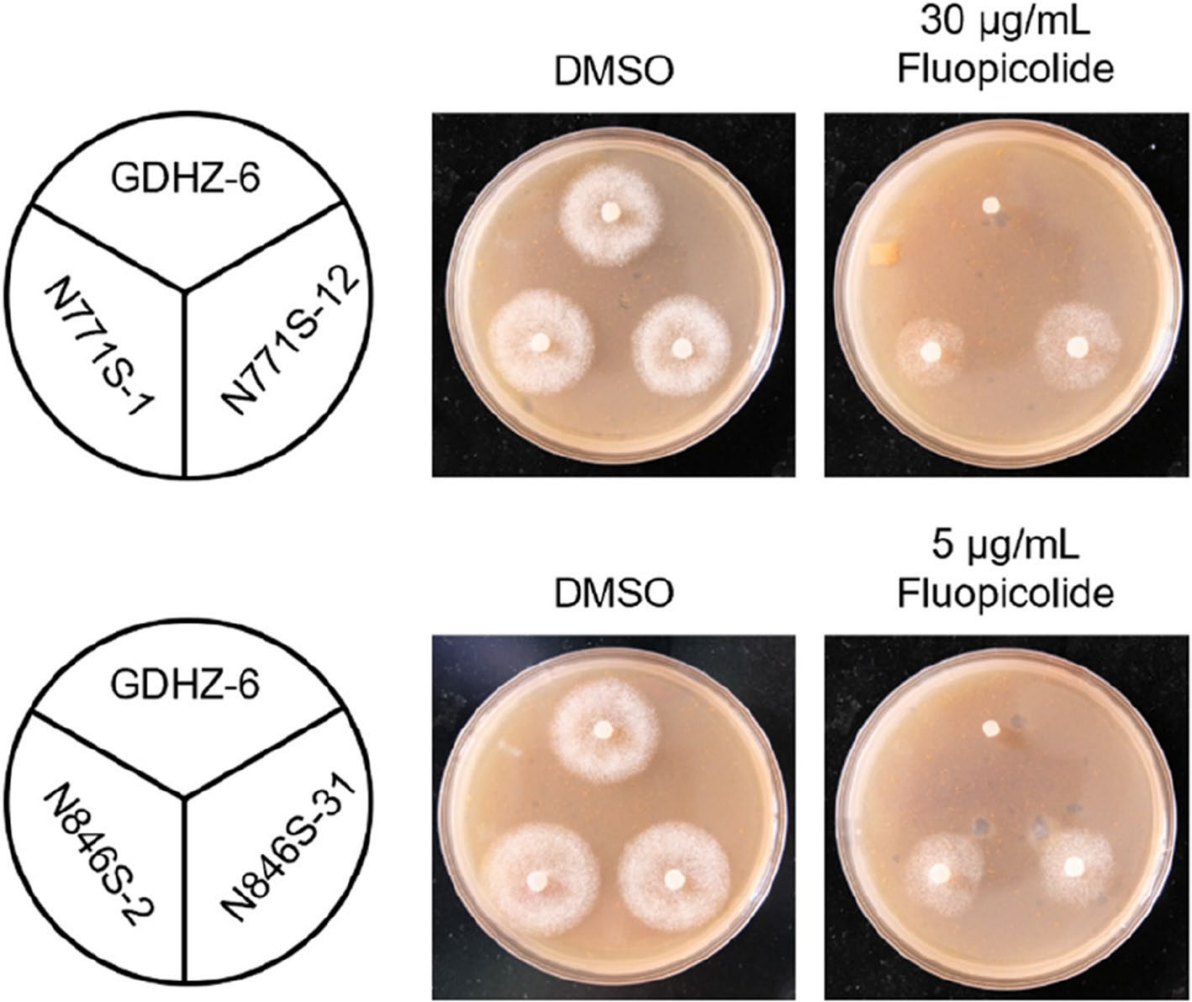
The sensitivity of overexpressing transformants to fluopicolide. Mycelial growth rate of overexpressing transformants on fluopicolide-amended V8 medium

Four overexpressing transformants of *PlVHA-a* with N771S and N846S point mutations were obtained by PEG/CaCl_2_-mediated protoplast transformation (Fig. 4). The fluopicolide sensitivity of transformants exhibited an EC_50_ ranging from 17.69 to 100 μg/mL and RF ranging from 147 to 833 (Table S3).

**Fig. 5:**
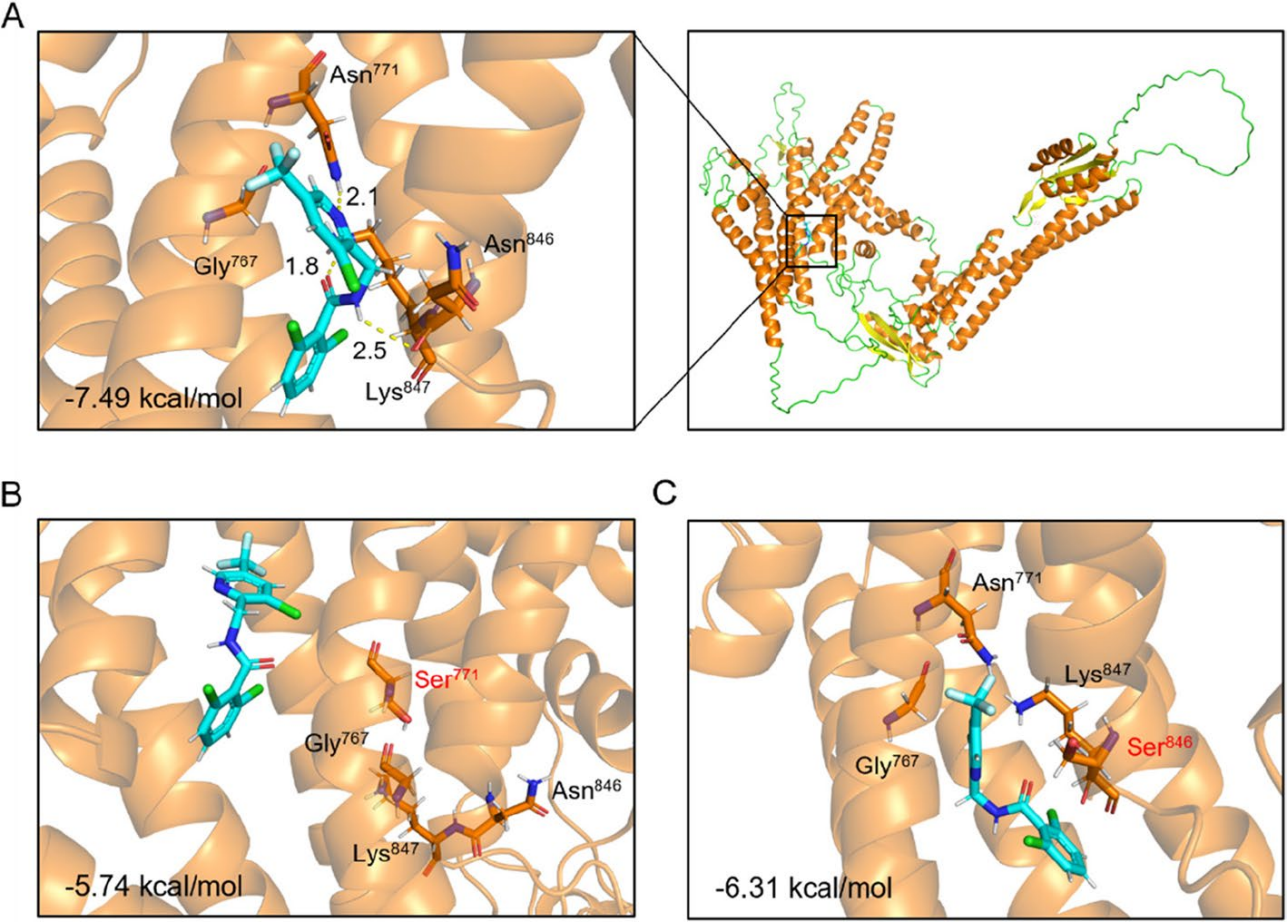
Docking of fluopicolide in the homology model binding pockets of PlVHA-a. **A** The binding of fluopicolide to PlVHA-a. **B** The binding of fluopicolide to PlVHA-a^N771S^. **C** The binding of fluopicolide to PlVHA-a^N846S^

Fluopicolide establishes three hydrogen bonds (H-bonds) with residues N771, N846, and K847 on PlVHA-a, resulting in a binding energy of − 7.49 kcal/mol (Fig. 5A). However, the introduction of S771 and S846 into the PlVHA-a model led to the loss of these critical hydrogen bonds, accompanied by a reduction in binding energies to − 5.74 kcal/mol and − 6.31 kcal/mol, respectively (Fig. 5B and C). These findings suggest that the N771S and N846S point mutations in PlVHA-a can confer resistance of *P. litchii* to fluopicolide.

## Discussion

Fluopicolide exhibits excellent efficacy in controlling oomycete diseases, effectively inhibiting zoospore release, sporangia germination, mycelial growth, and other developmental stages of oomycetes (Jackson et al. 2010). With an average EC_50_ value of 0.131 μg/mL across 125 *P. litchii* isolates collected from various regions in China, fluopicolide demonstrated remarkable effectiveness against *P. litchii*. The baseline sensitivity displayed a unimodal distribution, indicating the absence of resistant subpopulations in the field. This baseline can serve as a benchmark for monitoring variations in *P. litchii* sensitivity to fluopicolide.

Four resistant mutants of *P. litchii* were generated through fungicide adaptation, with RFs exceeding 600. By transferring the mutants onto fungicide-free V8 medium for ten generations, we showed that resistance can be stably inherited. The findings suggested that high-resistance mutants are more readily produced than low-resistance mutants and that stable resistance facilitates the dominance of these resistant populations. While the optimal temperature for both resistant mutants and their parental strains remained unchanged, biological characteristics such as sporangia production and germination rates were lower in mutants compared to in their parental strains. Cross-resistance experiments indicated positive cross-resistance between fluopicolide and fluopimomide, negative cross-resistance between fluopicolide and azoxystrobin, but no cross-resistance with other fungicides exhibiting different modes of action (metalaxyl, dimethomorph, oxathiapiprolin). Although the change in EC_50_ values was minimal, there was evidence of weak negative cross-resistance. Compared to the V-ATPase of wild-type isolates, the V-ATPase of mutants may require a greater consumption of ATP to sustain normal growth. In turn, azoxystrobin, a mitochondrial respiratory electron transport chain compound III inhibitor, inhibits ATP production, resistant strains with inhibited ATP synthesis are more sensitive to azoxystrobin. This might be the cause for the weak negative cross-resistance between the two fungicides. Nevertheless, the specific mechanism still requires further investigation. Under natural conditions or without exposure to pyridinylmethyl-benzamide fungicides, it is challenging for resistant mutants to become dominant populations. Implementing fungicide rotation strategies can effectively delay the emergence of fluopicolide-resistant phenotypes within field settings. In practical field situations, fluopicolide should be employed in combination with other fungicides with distinct modes of action, such as metalaxyl, dimethomorph, oxathiapiprolin, and azoxystrobin, with a particular emphasis on the combination of azoxystrobin and fluopicolide. In China, fluopicolide is not registered for the control of *P*. *litchii* as a single application; instead, it is registered in combination with fosetyl-aluminium for the control of *P*. *litchii*. This might be one of the principal reasons why no *P*. *litchii* resistant to fluopicolide has been discovered yet. In future, if the resistance of *P. litchii* to fluopicolide still emerges, other oomycete inhibitors need to be utilized instead of fluopicolide for treatment. Only when the resistance vanishes can it be reused for controlling oomycete diseases. In summary, the challenges associated with obtaining mutants under laboratory conditions, the CFI values obtained, and the inherent risks related to pathogen–fungicide interactions, suggest a moderate risk of *P. litchii* developing resistance against fluopicolide.

A recent report on the VHA-a protein in *P. capsici* indicated that four point mutations (G767E, N771Y, N846S, and K847R) can cause resistance to fluopicolide (Dai et al. 2024a). In *P. nicotianae*, it has been observed that G765E (corresponding to G767E in *P. litchii*) and N769Y (corresponding to N771Y in *P. litchii*) point mutations can confer resistance to fluopicolide (Liu et al. 2024). Similarly, in *P. infestans*, G755R/E point mutations (corresponding to G767R/E in *P. litchii*) are associated with resistance to fluopicolide (Peyrard et al. 2024). Furthermore, *Plasmopara viticola* has shown resistance against fluopicolide due to the G759N point mutation (corresponding to G767N in *P. litchii*) (Peyrard et al. 2024). In the case of *P. litchii*, it was found that N771S and K847N point mutations can cause resistance against fluopimomide (Dai et al. 2024b). Our research found that both N771S and N846S point mutations are capable of conferring resistance against fluopicolide in *P. litchii* as well.

The fluopicolide-resistant point mutations produced in *P. litchii* differed from those produced in different pathogens. For instance, *P. capsici* did not exhibit G767R/N, N771S, or K847N point mutations, and *P. litchii* did not show G767R/E/N, N771Y, or K847R point mutations. It may be necessary to increase the sample size to verify this phenomenon. Furthermore, the same type of point mutation resulted in different levels of fungicide sensitivity in different species. Specifically, the N846S point mutation led to low resistance to fluopicolide in *P. capsici* but high resistance in *P. litchii*, despite a protein similarity of over 97% between the two strains (Dai et al. 2024a, b). These differences in fungicide sensitivity may be attributed to specific amino acid variances.

Through genetic transformation experiments on *P. litchii*, four transformants were obtained and exhibited reduced sensitivity to fluopicolide with RF values ranging from 147 to 833. This suggests that both N771S and N846S point mutations can confer resistance to fluopicolide in *P. litchii.* However, it is worth noting that the RF values of mutants obtained through fungicide adaptation exceeded 600, while some of the transformants had RF values ranging from 147 to 253—significantly lower than those of mutants. The fluopicolide-resistance overexpressing transformants was readily lost, resulting in an unstable inheritance of the phenotype. This may be because these strains also possessed a set of *VHA-a* genes without any point mutations in their genome, which may result in lower expression levels compared to PlVHA-a mutants. In future studies, CRISPR/Cas9 technology should be used for site-specific mutations in *P. litchii*, thereby obtaining more accurate resistance data. The molecular docking results indicated that N771, N846, and K847 are key amino acids for binding fluopicolide to PlVHA-a as they form hydrogen bonds with this protein. However, introducing the N771S and N846S point mutations into PlVHA-a disrupted these hydrogen bonds and decreased the binding energy. The above results indicate that the two point mutations on PlVHA-a lead to resistance to fluopicolide in *P. litchii*.

## Conclusion

Fluopicolide demonstrates a good inhibitory effect on *P. litchii* with an average EC_50_ of 0.131 μg/mL. *P. litchii* has a moderate risk of developing resistance to fluopicolide, particularly through the N771S or N846S point mutations on PlVHA-a, which confer resistance to fluopicolide. This research provides an important theoretical basis for controlling litchi downy blight and for the appropriate use of fluopicolide in the field.

## Materials and methods

### Fungicides

Fluopicolide (97.2% active ingredient [a.i.], Bayer Crop Science, Shanghai, China), metalaxyl (96.0% a.i., Sigma-Aldrich Shanghai Trading Co. Ltd., Shanghai, China), dimethomorph (97.5% a.i., Frey Agrochemicals Ltd., Jiangsu, China), oxathiapiprolin (98.8% a.i., Du Pont Crop Protection, Wilmington, USA), azoxystrobin (98.5% a.i., Syngenta Biotechnology Co., Ltd., Shanghai, China), and fluopimomide (97.8% a.i., Shandong Sino-Agri United Biotechnology Co., Ltd., Shandong, China) were used to determine the sensitivity of *P. litchii* strains. The fungicides all had technical-grade purity, were diluted to 10^4^ µg/mL using dimethyl sulfoxide, and stored at 4 °C.

### *P*. *litchii* isolates and culture medium

A total of 125 *P. litchii* isolates were collected from the Chinese provinces of Fujian, Guangxi, Guangdong, and Hainan (Table S1). These isolates were identified by internal transcribed spacer (ITS) region sequencing (White et al. 1990; Fu et al. 2024). Strains were cultured using 10% V8 medium, which contained 100 mL of V8 juice, 1.4 g calcium carbonate, and 15 g of agar, with deionized water added to form a final volume of 1 L.

### Baseline sensitivity of *P. litchii* to fluopicolide

The sensitivity of 125 strains to fluopicolide and the sensitivity of mutants to other fungicides (metalaxyl, dimethomorph, oxathiapiprolin, azoxystrobin, fluopimomide) were measured using the mycelium growth rate method, and the median effective concentration (EC_50_) values were obtained according to previously described method (Li et al. 2020; Fei et al. 2023). The strains were cultured at 25℃ for 5 d, and then 5-mm mycelial plugs were transferred to the V8 plates with a gradient concentration of fluopicolide or other fungicides.

### Generation of *P. litchii* mutants resistant to fluopicolide

Seven wild-type strains (GDHZ-3, GDHZ-6, HN10-1, HN10-2, FJZZ-1, FJFZ-12, and FJFZ-34) were selected for fluopicolide adaptation. The strains were transferred to V8 medium and cultured in the dark at 25℃ for 5 d. The 5-mm mycelial plugs were attached to V8 medium plates containing 1 µg/mL fluopicolide side down and pre-cultured at 25℃ for 10 d. After the new mycelium filled the plates, new mycelial plugs were transferred to a fungicide-free V8 plate for expanded culture and then transferred to a new fluopicolide-amended medium with progressively increasing concentrations of fluopicolide (2.5, 5, 10, 20, 40, 80, and 160 µg/mL). Resistance stability was determined by continuously transferring mycelia to fungicide-free V8 medium plates for 10 generations. The *PlVHA-a* gene was amplified using previously developed primers (F: 5′-TGAAGTGGAGTATTGACTGGAC-3′ and R: 5′-GAAATGGAGAAGAAAGCGG-3′) (Dai et al. 2024b).

### Characterization of *P. litchii* isolates

#### Effect of temperature on *P. litchii* mycelial growth

The fluopicolide-resistant mutants and their parental strains were incubated on V8 media at 4, 13, 18, 22, 25, 28, and 30 °C while monitoring colony diameters. Each treatment contained three replicates, and the whole experiment was repeated three times.

#### Sporangia formation, sporangia germination, zoospore generation, and cystospore germination in vitro

The resistant mutants and parental strains of *P. litchii* were transferred to V8 medium and cultured under 25℃ for 5 d in the dark. Twenty-five 5-mm-diameter plugs were placed in 4 mL sterile water and shaken for 50 s to obtain sporangia suspension.

The sporangia concentrations of each resistant mutant and their parental strain were measured under optical microscopy using a microscope slide. Sporangia suspensions of 200 μL were uniformly coated on a 1.5% agar plate and cultured at 25℃ for 10 h. Two hundred and fifty sporangia were counted under an optical microscope, and the sporangium germination rates of each resistant mutant and parental strain were calculated. Sporangia suspensions were cultured at 15℃ under intense light for 2 h to release zoospores, 150 sporangia were counted under an optical microscope, and the percentage of empty sporangia was calculated. Zoospore suspensions of 200 μL were uniformly coated on a 1.5% agar plate and cultured at 25℃ for 10 h. Two hundred and fifty cystospores were counted under an optical microscope. Each treatment contained three replicates, and the whole experiment was repeated three times.

#### Virulence on litchi fruit

Fresh litchi fruit (cultivar: Guiwei) were sterilized for 8–10 min using 0.5% NaClO, and then washed 3–5 times with sterilized water. The resistant mutants and parental strains of *P. litchii* were cultured for 5 d at 25 °C. Mycelial plugs were attached to the center of the litchi fruit and cultured at 25℃ and 80% relative humidity for 4 d in the dark. Each treatment contained six replicates, and the whole experiment was repeated three times. The lesion outline was drawn on the surface of each litchi with a plastic sheet, and the lesion area was calculated with ImageJ software.

### Transformation of *P. litchii*

Using the homologous recombination method (Hieff Clone® Plus Multi One Step Cloning Kit, Yeasen Biotechnology Co., Ltd. Shanghai, China), the *PlVHA-a* gene containing the N771S or N846S point mutation was linked into the expression vector pTOR-3 × FLAG, and the enzyme restriction sites were *Nhe*I and *Sac*II. The vector was transferred into the cells of wild-type strain GDHZ-6 by protoplast transformation (Fang and Tyler 2016). Primers used for vector construction are shown in Table S2.

### Molecular docking

The relationship between the point mutation in the PlVHA-a protein and fluopicolide-resistance was further validated using molecular docking. The protein model used in this study was consistent with previously reported protein models (Dai et al. 2024b). YASARA software was connected to AutoDock 4.2.6 for molecular docking simulation. Fluopicolide was used as a ligand for docking with PlVHA-a without point mutations and PlVHA-a with PlVHA-a^N771S^ and PlVHA-a^N846S^.

### Statistical analysis

Significant differences in colony diameter, sporangia formation, sporangia germination, zoospore generation, cystospore germination, lesion area, and compound fitness index (CFI) values were determined using one-way ANOVA and Tukey’s honest significant difference test (*P* < 0.05) with Data Processing System software (version 6.50).

## Supplementary Information

Supplementary Material 1: Table S1. Field strains for baseline sensitivity of *P. litchii* to fluplicolide. Table S2. Primers employed for expressing vectors of PlVHA-a. Table S3. Sensitivity of overexpressing transformants of *PlVHA-a* with N771S and N846S point mutations to fluopicolide. ^y^EC_50_, 50% inhibition of mycelial growth; ^z^RF, resistance factor = EC_50_ of transformation / EC_50_ of the paretal isolate.

## Data Availability

Data and materials will be made available on request.

## Abbreviations

EC_50_: Median effective concentration
V-ATPase: Vacuolar H^+^-ATPase
PlVHA-a: *Phytophthora**litchii* V-ATPase subunit a
LPB: Litchi downy blight
CFI: Compound fitness index
ITS: Internal transcribed spacer
