# Supplementary material for: The resistance risk of fluopicolide and resistance-associated point mutations in the target protein PlVHA-a in Phytophthora litchii
Source: Stress Biol. 2025 Apr 21;5(1):26. doi: 10.1007/s44154-025-00218-9 (PMC12011701; doi:10.1007/s44154-025-00218-9)
Supplement: Supplementary file 1 — Supplementary Material 1: Table S1. Field strains for baseline sensitivity of P. litchii to fluplicolide. Table S2. Primers employed for expressing vectors of PlVHA-a. Table S3. Sensitivity of overexpressing transformants of PlVHA-a with N771S and N846S point mutations to fluopicolide. yEC50, 50% inhibition of mycelial growth; zRF, resistance factor = EC50 of transformation / EC50 of the paretal isolate. [file 44154_2025_218_MOESM1_ESM.docx]

**Table S1** Field strains for baseline sensitivity of *P. litchii* to fluplicolide.

**Table S2** Primers employed for expressing vectors of PlVHA-a.

**Table S3** Sensitivity of overexpressing transformants of *PlVHA-a* with N771S and N846S point mutations to fluopicolide. ^y^EC_50_, 50% inhibition of mycelial growth; ^z^RF, resistance factor = EC_50_ of transformation / EC_50_ of the paretal isolate.

**Table S1**

| Isolates | Province | Detailed | Amount | Time |
| --- | --- | --- | --- | --- |
| FJZZ1-8 | Fujian | Baihua Village, Jiuhu Town, Zhangzhou City | 7 | 2019.6.20 |
| FJZZ9 |  | Shanmei Village, Tianbao Town, Zhangzhou City | 1 | 2019.6.20 |
| FJZZ18-20 |  | Guanbei Town, Zhao'an County, Zhangzhou City | 3 | 2019.6.20 |
| FJPT21-26 |  | Litchi Park, Chengxiang District, Putian City | 5 | 2019.6.22 |
| FJPT27-30 |  | Xiahengshan Village, Xindu Town, Putian City | 4 | 2019.6.22 |
| FJFZ31-44 |  | Xujia Village, Minhou County, Fuzhou City | 12 | 2019.6.25 |
| FJFZ45-59 |  | Forest Park, Jin'an District, Fuzhou City | 13 | 2019.6.25 |
| FJND60-68 |  | Erdushang Village, Feiluan Town, Ningde City | 9 | 2019.6.30 |
| FJND69-74 |  | Erduxia Village, Feiluan Town, Ningde City | 6 | 2019.6.30 |
| FJ10-1 |  | Ningde City | 1 | 2010.6 |
| GXBL1-34 | Guangxi | Tangjiao Village, Beiliu Town, Beiliu City | 28 | 2019.7.8 |
| GXQZ-2 |  | Qinzhou Suburb | 1 | 2019.7.1 |
| GXXW22-25 |  | Xiawan Town, Guiping City | 4 | 2019.7.4 |
| GX10-1 |  | Beiliu City | 1 | 2010.6.8 |
| HN10-1 | Hainan | Haikou City | 1 | 2010.6.2 |
| HN10-2 |  |  | 1 |  |
| HN10-3 |  |  | 1 |  |
| HK-1 |  |  | 1 |  |
| GDHZ1-13 | Guangdong | Huizhou City | 13 | 2019.6.5 |
| GDSZ1-13 |  | Shenzhen City | 13 | 2019.6.5 |

**Table S2**

| **Primer** | **Sequence (5'→3')** |
| --- | --- |
| 3FPl-aF | CCTTGAGGTTGCTAGCATGAAGTGGCTCCGCTCGG |
| 3FPl-aR | CACCCCGCGGTCTAGACTAGGGCTGCTGCGAGTCC |
| 3FPl846F1 | GGATCCCTTGAGGTTGCTAGCATGAAGTGGCTCCGCTCGG |
| 3FPl846R1 | GTAGAACTTGCTCTGGAACTCCACCCAGTGC |
| 3FPl846F2 | AGTTCCAGAGCAAGTTCTACAAAGCTGACG |
| 3FPl846R2 | GCGACGAAAACCGATTTGTTAACCAAATTAAATAAGTCGATATACAGA |
| 3FPl771F1 | GGATCCCTTGAGGTTGCTAGCATGAAGTGGCTCCGCTCGG |
| 3FPl771R11 | CGATGCCGTGCTCGAGACCATCCCCAGCAC |
| 3FPl771F22 | ATGGTCTCGAGCACGGCATCGTACCTGCGT |
| 3FPl771R2 | GCGACGAAAACCGATTTGTTAACCAAATTAAATAAGTCGATATACAGA |

**Table S3**

| Strain | Origin | EC_50_^y^ | RF^z^ |
| --- | --- | --- | --- |
| GDHZ-6 | Parent | 0.12 | - |
| 771-1 | Transformation | 79.78 | >664 |
| 771-12 | Transformation | >100 | >833 |
| 846-2 | Transformation | 30.40 | >253 |
| 846-31 | Transformation | 17.69 | >147 |
